# Gel shift experiments with fragments of the *Trypanosoma brucei* RNA-binding protein RBP10

**DOI:** 10.1186/s13104-022-06143-8

**Published:** 2022-07-15

**Authors:** Bin Liu, Christine Clayton

**Affiliations:** 1 Heidelberg University Centre for Molecular Biology (ZMBH), Heidelberg, Germany; 2 Present Address: Hebei Viroad Biotechnology Co. Ltd, Shijiazhuang, China

**Keywords:** *Trypanosoma brucei*, RNA Recognition Motif, RNA binding, Developmental regulation

## Abstract

**Objective:**

*Trypanosoma brucei* is a parasite of mammals and Tsetse flies, and control of mRNA stability is critical for parasite survival in the two different hosts. *T. brucei* RBP10 is a protein with a single RNA Recognition Motif (RRM) which is expressed only in the mammalian (bloodstream) form. Numerous observations suggest that RBP10 binds to procyclic-specific mRNAs and targets them for destruction, and there is also some evidence for selective binding of RBP10 to RNAs containing the motif UA(U)_6_. We here investigated this binding further.

**Results:**

We tested in vitro binding of RBP10 to two different probes in solution. One contained two copies of UA(U)_6_, and the other two copies of a mutant version, UACUCUCU, which is inactive in regulation. An N-terminal segment of RBP10, including the RRM domain and 90 residues to its C-terminus, could be produced as soluble protein. This could bind both probes in vitro with similar affinities in the low micromolar range, which is not atypical for a single RRM. Soluble RBP10 therefore did not distinguish between UA(U)_6_ and UACUCUCU. Since no other sequences were tested, the requirements for RBP10 RNA binding remain to be determined.

## Introduction

The RNA Recognition Motif (RRM) is a sequence of approximately 90 residues which interacts with single-stranded with RNA or DNA [1]. In some proteins, the RRM domain has also been shown to interact with other proteins [1]. A single RRM can bind 3–5 nt with affinities that range from the nM to the high µM range [1, 2]. Higher RNA-binding sequence specificities and affinities are achieved by cooperativity between several RRM domains, or between RRMs and other RNA-binding motifs [1–3].

*Trypanosoma brucei* is a parasite of mammals and Tsetse flies, and control of mRNA stability is critical for parasite survival in the two different hosts. *T. brucei* RBP10 is a protein with a single RNA Recognition Motif (RRM) which is expressed only in the mammalian (bloodstream) form. Numerous observations, including the effects of RBP10 depletion in bloodstream forms and of inappropriate expression in the procyclic (Tsetse fly midgut) form, and the results of pull-down followed by RNA-Seq, suggest that RBP10 binds to procyclic-specific mRNAs and targets them for destruction [4, 5].

The 3′-untranslated regions (3′-UTRs) of the RBP10-bound mRNAs were strongly enriched in the motif UA(U)_6_. The role of this motif in causing mRNA instability had previously been demonstrated in the mRNAs encoding EP procyclin, a major surface protein of procyclic forms [6, 7], and in some other procyclic-specific mRNAs [8]. Reporters expressing chloramphenicol acetyltransferase (CAT) or another open reading frame with the *EP1* 3′-UTR [6, 7] were developmentally regulated, and a single-stranded 26mer region [9] containing two copies of the UA(U)_6_ motif was required for the regulation [6, 7]. After immunoprecipitation of RBP10 from cells expressing CAT reporters with or without the 26mer, CAT mRNA was preferentially found only if the 26mer was present [4].

The observations described above suggested that RBP10 binds specifically to the sequence UA(U)_6_, but it is difficult to see how this could be possible since there is only a single RRM and no evidence for interaction with other RNA-binding proteins. We here further investigated the binding of RBP10 to RNA.

## Main text

### Protein expression

**Fig. 1:**
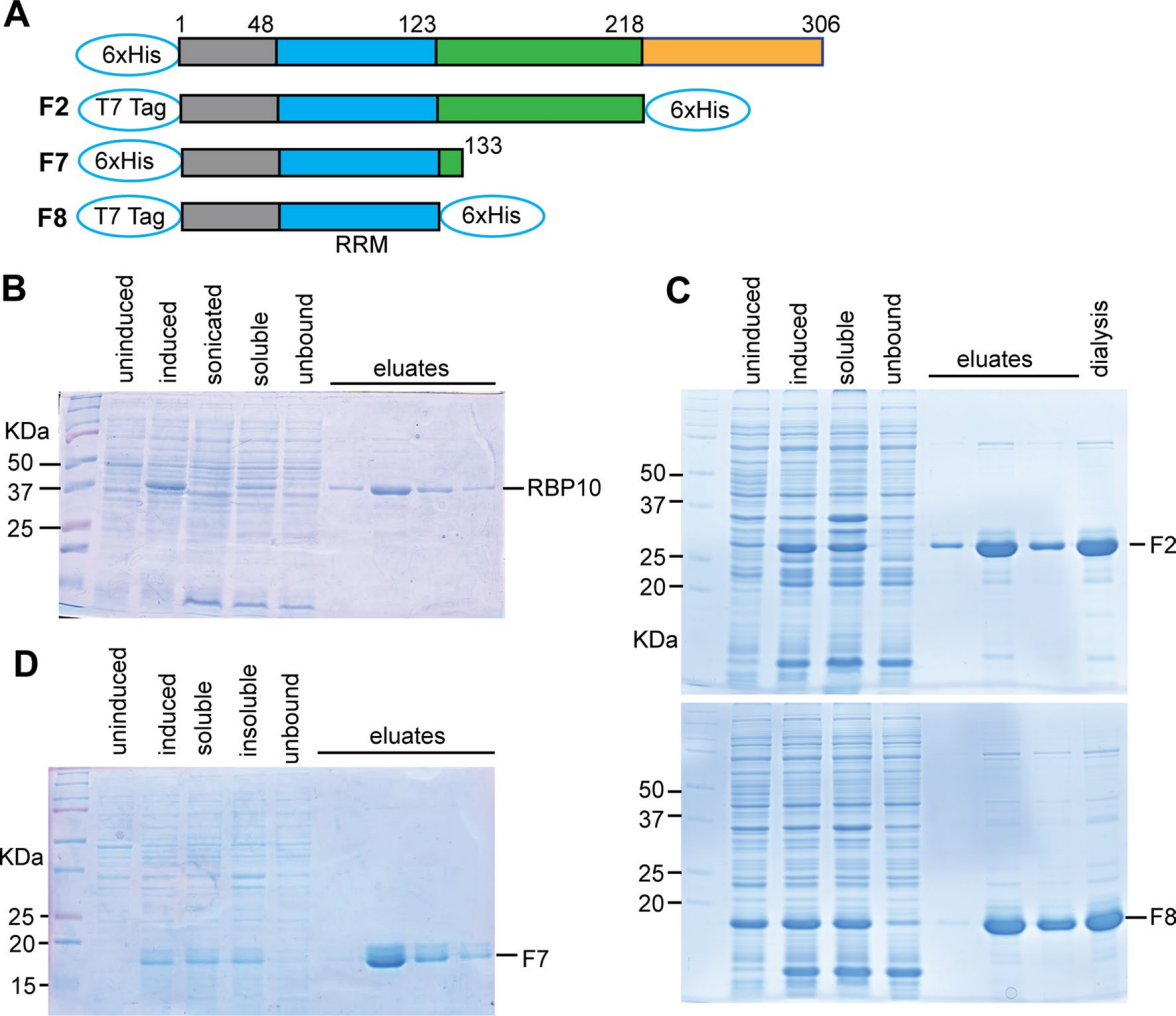
Production of recombinant proteins. **A** Cartoon showing RBP10 and produced fragments. F2 and F8 were expressed from pET24a ( +) and F7 from pQEA38. **B** Coomassie-blue stained SDS-polyacrylamide gel showing purification of His-RBP10. Equal proportions of lysates from induced and uninduced cells, sonicated extracts and the soluble and insoluble fractions, are shown. Equal volumes of eluate fractions are also shown. **C** Purification of F2 and F8 proteins. Details are as in **B** except that samples from dialyzed eluates are also illustrated. **D** Purification of F7 protein. Details are as in **B**

BL21 *E. coli* were transformed with the plasmids expressing proteins illustrated in Fig. 1. Expression was induced in 100 ml culture, with IPTG (1 mM final concentration) at 37 °C for 3 h. Cells were pelleted, washed with binding buffer (50 mM NaH_2_PO_4_, 300 mM NaCl, 10 mM imidazole, 10% glycerol, pH 8.0) then resuspended in 5 ml binding buffer with 1 mM PMSF and protease inhibitor cocktail without EDTA. Lysozyme was added to a final concentration of 1 mg/ml. All subsequent steps were at 4 °C. The cells were sonicated, then the lysate was centrifuged at 13000*g* for 20 min. The supernatant was added to 500 µl nickel beads, and protein allowed to bind at 4 °C for 1.5 h with mixing. The beads were then transferred to an empty column, and washed 3 times with 50 mM NaH_2_PO_4_, 300 mM NaCl, 20 mM imidazole, pH 8.0. Elution was with 50 mM NaH_2_PO_4_, 300 mM NaCl, 250 mM imidazole, pH 8.0. Glycerol was added to a final concentration of 1%, and eluted samples were stored at − 70 °C. Sometimes proteins were dialyzed against 10 mM Tris (8.0), 50 mM NaCl and 10% glycerol, using a 6–7 kDa pore membrane.

We first attempted to express and purify full-length RBP10 (Fig. 1A). Although the protein could be expressed, as described previously [5], the amounts were relatively low and sonication appeared to reduce the amount (Fig. 1B). Some soluble RBP10 could be obtained (Fig. 1B) but it precipitated after freeze-thawing and upon dialysis. Purification of RBP10 fragments without the C-terminus was rather more successful (example in Fig. 1C). The tags were not removed so they could have affected the subsequent results.

### RNA gel shifts

RNA gel shifts were done as described in [10]. To label RNA oligonucleotides, 50 pmol RNA were incubated in a 50 µl reaction at 37 °C for 30 min with 20 units T4 polynucleotide kinase (New England Biolabs) and 50 µCi gamma-[^32^P]-ATP in the buffer provided by the manufacturer. The labelled probes were purified using a nucleotide removal kit. The yield was assumed to be 80%. For RNA–protein binding, probes were incubated with protein in 20 mM Tris pH8, 50 mM NaCl, 1 mM DTT, 0.1 mg/ml tRNA, 10 µg/ml heparin, 0.01% IGEPAL C-630, with a final volume of 20 µl (probe concentration 500 pM). 5% non-denaturing polyacrylamide gels (Tris-borate-EDTA) were pre-run at 85 V for 1 h in 0.5 × TBE before sample loading.

**Fig. 2:**
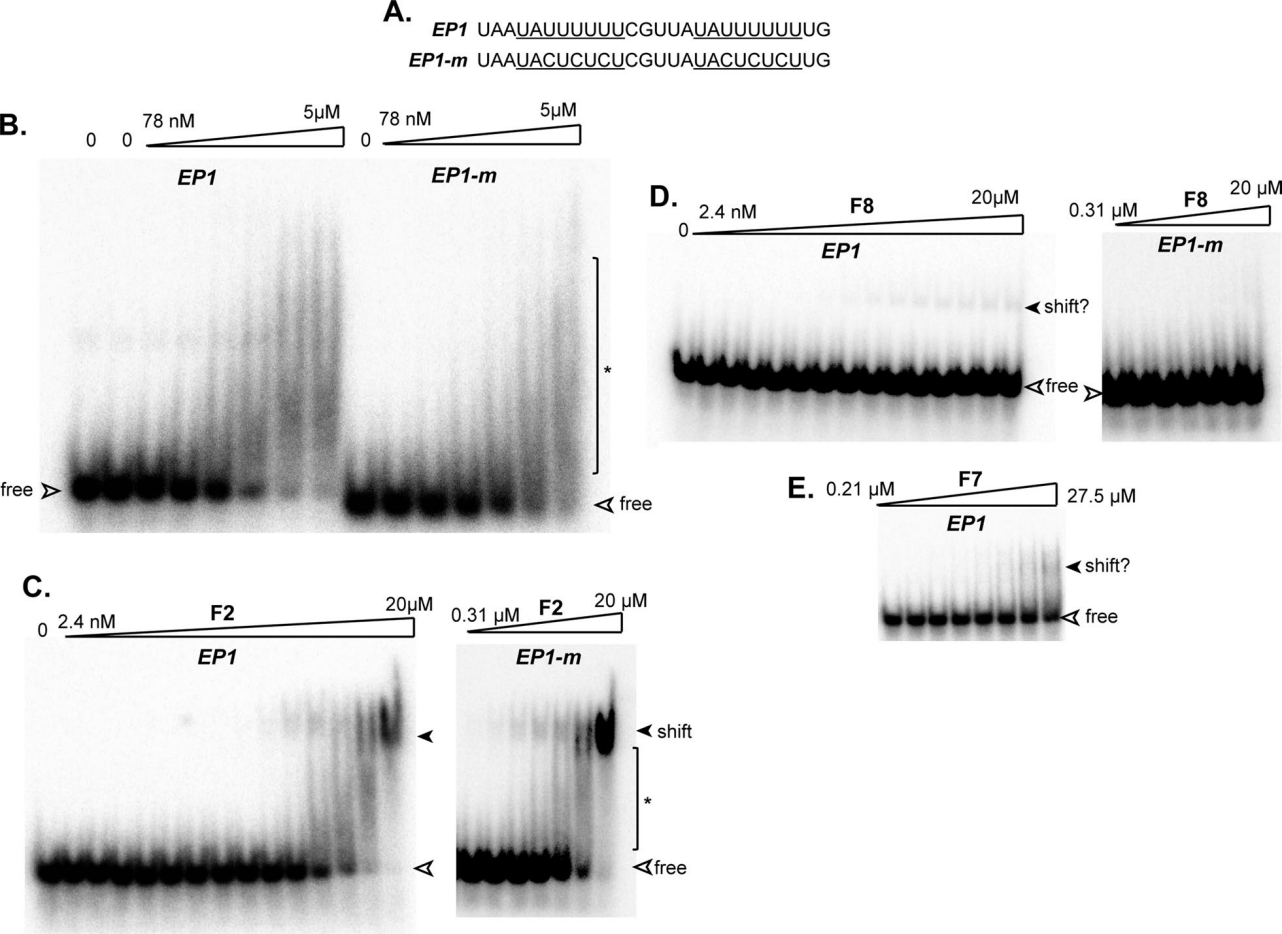
Gel shifts obtained using purified RBP10 and fragment F2. **A** Sequences of the 26mer probe (*EP1*) and the mutant version (*EP1-m*), which is inactive in developmental regulation, both used at 500 pM. **B** Gel shift with full-length RBP10, added in progressive twofold dilutions. The asterisk shows a smear of bound RNAs. **C** Gel shift with RBP10 fragment F2, added in progressive twofold dilutions. **D** Gel shift with RBP10 fragment F8, added in progressive twofold dilutions. **E** Gel shift with RBP10 fragment F7, added in progressive twofold dilutions

We measured binding of the various RBP10 preparations with two probes: the 26mer that is required for stability of the mRNA encoding EP procyclin, and a mutant version that does not give regulation [6] (Fig. 2A). Full-length RBP10 with the wild-type probe gave a higher molecular weight smear (asterisk) at a concentration of about 600 nM, but no clear band was obtained (Fig. 2B). Moreover, the results with the mutant probe were very similar. It is possible that the protein was aggregating during the procedure.

**Fig. 3:**
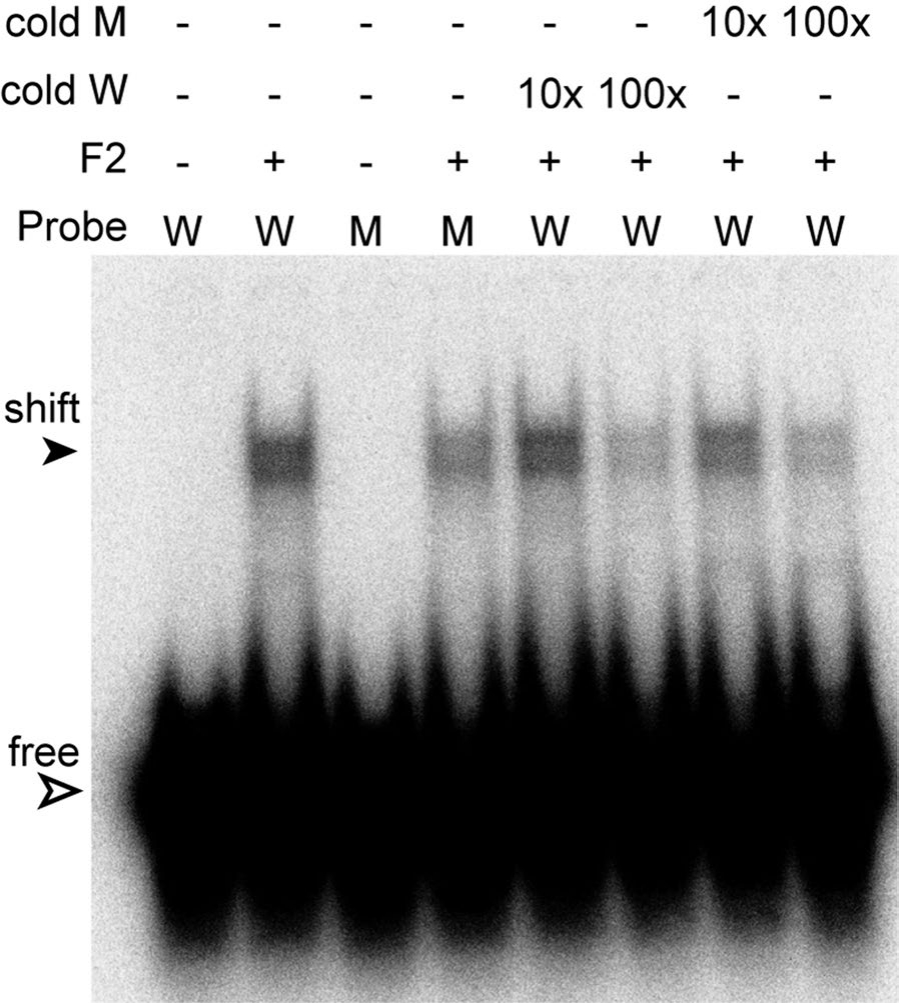
Lack of specific competition. Gel shifts were done with RBP10 fragment F2 (500 nM), using mutant (M) or wild-type (W) probes (5 nM), in the presence or absence of un-labelled competitor RNAs

We next tested various fragments (Fig. 1A). The only reproducible shifts were obtained with fragment F2, which gave a clear shifted band (Fig. 2C). There was again no specificity for the wild-type 26mer and the dissociation constant was in the low micromolar range. Addition of cold competitor RNA also revealed no specificity (Fig. 3). Fragments F7 and F8 gave only weak shifts at 20–30 µM (a decrease in association constant of more than tenfold, Fig. 2D, E). This suggests that the 90 C-terminal residues in fragment F2 play a role either in RRM domain folding, or in RNA binding. Since the action of RRMs is often cooperative, we also tried using a dimer of F2, with a linker (GGGGSx3) between the copies, but rather surprisingly, this behaved like fragments F7 and F8 (not shown).

### RNA pull-downs

For in vivo pull-downs, we used bloodstream forms in which all RBP10 bore an N-terminal tandem affinity purification (TAP) tag at the N-terminus (TAP-RBP10). Since RBP10 is essential, and the cells grew normally, the tagged version must have been functional: otherwise, cells expressing only the tagged version would not have been viable. We used cells that had chloramphenicol acetyltransferase (CAT) reporters integrated into the alpha–beta tubulin locus, so were transcribed by RNA polymerase II. The reporters expressed *CAT* mRNAs with various 3′-UTRs. These were the *EP1* wild-type 3′-UTR and a version lacking the regulatory 26mer; the *PGKB* 3′-UTR, which also has two copies of UA(U)_6_, and a deleted version [11]; and as an additional control, the actin 3′-UTR. Endogenous tubulin mRNA served as an internal control. After UV irradiation, cells were lysed, and TAP-RBP10 was purified via the IgG-binding domain then released with Tobacco Etch protease, exactly as previously done for RNA-Seq [4]. RNAs were detected by reverse transcription and PCR, with different numbers of PCR cycles, followed by gel electrophoresis [4]. Although specific precipitation was sometimes detected, results were insufficiently reproducible for detailed investigation. We were also unable find primers for quantitative PCR of *CAT* that gave satisfactorily specific signals. Problems included the low expression levels of the reporter mRNAs with wild-type *EP1* and *PGKB* 3′-UTRs relative to mutant versions or the *ACT* 3′-UTR, and RNA degradation.

## Limitations

We here showed that an N-terminal segment of RBP10 that includes the 48 N-terminal residues, the RRM domain, and 85 further residues towards the C-terminus, could be produced as soluble protein, and could bind two pyrimidine-rich RNAs in vitro. The binding affinity was well within the range expected for a single RRM, but the purified proteins did not distinguish between a sequence that is active in developmental regulation in vivo, and one that is inactive. A major limitation was that we tested in vitro binding with only two RNA sequences, so we do not know whether RBP10 might also bind purine-rich RNA. Proteins with only 10 residues, or no residues, C-terminal to the RRM had at least tenfold lower affinities for the probes, suggesting that at least part of the region from residues 133–218 either directly contributes to RNA binding, or is important for RRM-domain folding.

The results of TAP-RBP10 pull-downs from cells expressing CAT reporters were irreproducible. More conclusive results—to either confirm, or disprove, the sequence specificity of RBP10 mRNA binding—might be obtained by using an RNA polymerase I promoter to drive expression of the reporter mRNAs, and an open reading frame with reliable real-time PCR primers for quantitation.

## Data Availability

Further information and plasmids are available from the corresponding author.

## Abbreviations

RRM: RNA Recognition Motif
TAP: Tandem affinity purification
